# Inborn errors of immunity—recent advances in research on the pathogenesis

**DOI:** 10.1186/s41232-021-00159-6

**Published:** 2021-03-25

**Authors:** Motoi Yamashita, Kento Inoue, Tsubasa Okano, Tomohiro Morio

**Affiliations:** Department of Pediatrics and Developmental Biology, Graduate School of Medical and Dental Sciences, Tokyo Medical and Dental University (TMDU), 1-5-45 Yushima, Bunkyo-ku, Tokyo, 113-8510 Japan

**Keywords:** Inborn errors of immunity, Next-generation sequencing, Functional validation

## Abstract

Primary immunodeficiency (PID) is a genetic disorder with a defect of one of the important components of our immune system. Classical PID has been recognized as a disorder with loss of function of the immune system. Recent studies have unveiled disorders with immune dysfunction with autoimmunity, autoinflammation, allergy, or predisposition to malignancy. Some of them were caused by an augmented immune function or a defect in immune regulation. With this background, the term inborn errors of immunity (IEI) is now used to refer to PID in the International Union of Immunological Societies (IUIS) classification. More than 400 responsible genes have been identified in patients with IEI so far, and importantly, many of them identified lately were caused by a heterologous mutation. Moreover, the onset is not necessarily in childhood, and we started seeing more and more IEI patients diagnosed in adulthood in the clinical settings. Recent advances in genetic analysis, including whole-exome analysis, whole-genome analysis, and RNA-seq have contributed to the identification of the disease-causing gene mutation. We also started to find heterogeneity of phenotype even in the patients with the same mutation in the same family, leading us to wonder if modifier gene or epigenetic modification is involved in the pathogenesis. In contrast, we accumulated many cases suggesting genetic heterogeneity is associated with phenotypic homogeneity. It has thus become difficult to deduce a responsible gene only from the phenotype in a certain type of IEI. Current curative therapy for IEI includes hematopoietic cell transplantation and gene therapy. Other curative therapeutic modalities have been long waited and are to be introduced in the future. These include a small molecule that inhibits the gain-of-function of the molecule- and genome-editing technology. Research on IEI will surely lead to a better understanding of other immune-related disorders including rheumatic diseases and atopic disorders.

## Background

Primary immunodeficiency (PID) is defined as inborn disorders in which a part of the immune system fails to function properly [1, 2]. The disease entity includes a variety of disorders, and PID exhibits protean complications. Since the immune system is essential in protection against bacteria, fungi, viruses, and other pathogens, the patients with PID often suffer from a wide range of severe infections. Though susceptibility to infection, immunocompromised status, has been characteristic in most PID patients, major symptoms of PID can be diverse. They include susceptibility to a narrow group of pathogens (i.e., Epstein-Barr virus, papillomavirus), autoimmunity, extremely high tendency to develop malignancy, severe allergy, and autoinflammation [3]. With these considered, the International Union of Immunological Societies (IUIS) currently uses “inborn errors of immunity” (IEI) for PID, and the expert committee on IEI has been continuing to update IEI classification every 2 years [1].

IEI now includes more than 400 different diseases caused by >400 different responsible genes [1]. It was not difficult for IEI physicians to memorize most of IEI diseases in the 1980s. This was partly because the majority of the disorders could be categorized into, and understood as, defects of B cells, T cells, neutrophils, monocytes, complements, or syndromic immunodeficiency with characteristic features. IEI is now more diverse, sometimes affecting both innate immunity and adaptive immunity or being caused by non-hematological cells.

We also learned the presence of genetic heterogeneity associated with physiological homogeneity, in which the patients exhibit a similar phenotype and yet the responsible genes are different. Mutation in one gene often leads to completely different phenotypes. This is often caused by the functional type of mutation, i.e., gain-of-function vs loss-of-function, and is also considered to be caused by gene variants of other related genes.

This review starts from the spectrum of IEI, genotype, and phenotype, moves to a brief introduction of the disorders mainly exhibiting autoimmunity or autoinflammation, and ends with the introduction of our current research interest.

## Main text

### 2019 IUIS IEI classification

2019 IUIS IEI classification added 64 new genetic defects that were reported in the past 2 years since 2017 [1]. The category of IEI classification is shown in Table 1.

**Table 1:** Classification of IEI (IUIS2019)^1^

	Category	Representative diseases	Example of newly added diseases
Table 1	Immunodeficiencies affecting cellular and humoral immunity	Severe combined immunodeficiency (SCID)	IKZF1 deficiency (dominant negative)
Table 2	Combined immunodeficiencies with associated or syndromic features	Wiskott-Aldrich syndrome (WAS) Hyper-IgE syndrome (HIE)	IL6 signal transducer (IL6ST) deficiency (HIE)
Table 3	Predominantly antibody deficiencies	X-linked agammaglobulinemia (XLA) Common variable immunodeficiency (CVID)	SLC39A7 (ZIP7) deficiency (B cell deficiency)
Table 4	Diseases of immune dysregulation	Autoimmune polyglandular syndrome (APS) Autoimmune lymphoproliferative syndrome (ALPS)	TGFB1 deficiency (colitis)
Table 5	Congenital defects of phagocyte number or function	Chronic granulomatous disease (CGD)	CYBC1 deficiency (CGD)
Table 6	Defects in intrinsic and innate immunity	Chronic mucocutaneous candidiasis disease (CMCD)	IRF4 deficiency, IRF9 deficiency (severe viral infection)
Table 7	Autoinflammatory disorders	Familial Mediterranean fever (FMF)	DNASE2 deficiency, OAS1 deficiency
Table 8	Complement deficiencies	Hereditary angioedema (HAE)	
Table 9	Bone marrow failure	Dyskeratosis congenita	
Table 10	Phenocopies of inborn errors of immunity	Ras-associated ALPS like disorder (RALD) Good syndrome	

Classical IEI includes immunodeficiencies affecting cellular and humoral immunity, combined immunodeficiency, as exemplified by such disease as X-linked severe combined immunodeficiency (SCID). Predominantly, antibody deficiencies include B cell deficiency (i.e., X-linked agammaglobulinemia) and common variable immunodeficiency (CVID).

Examples of non-classical IEI are the disorders categorized into “diseases of immune dysregulation,” “defects in intrinsic and innate immunity,” and “autoinflammatory disorders.” “Defects in intrinsic and innate immunity” include Mendelian susceptibility to mycobacterial disease (MSMD), epidermodysplasia verruciformis (EV: susceptibility to HPV), predisposition to severe viral infection, herpes simplex encephalitis (HSE), predisposition to invasive fungal diseases, predisposition to mucocutaneous candidiasis, TLR signaling pathway deficiency with bacterial susceptibility, other inborn errors of immunity related to non-hematopoietic tissues, and other inborn errors of immunity related to leukocytes. Forty-two known gene defects were listed in autoinflammatory disorders, in which 9 new gene defects were added in 2019.

The classification can be done from different angles/views. For example, if molecular pathogenesis was completely identified, IEI could be categorized in view of the molecular pathway. Interferonopathy [4] and tregopathy [5] are among such examples. We may use such categories as NFκB-pathy [6], ikarosopathy, JAK-STAT diseases [7], and so on in the future.

### Technology to identify pathogenic variant and to validate the pathogenicity in vitro

It is not unexpected that most of the new disease-causing variants were identified by massive parallel sequencing/next-generation sequencing (NGS) following the introduction of NGS into the gene-hunting field. The typical and standard approach to identify a pathogenic variant is to first employ panel sequencing, as a standard medical practice in Japan, and then to advance to whole-exome/genome analysis (WEA/WGA) when the causative gene was not identified. RNA-seq-based mapping and identification of mutations have also been employed to detect splice mutation, altered level of expression, translocation, and so on.

Usage of a pipeline to call a bona fide pathogenic variant is critical, and when the variant has not been reported, and is a single case, other strong supporting evidence should be accompanied. For instance, genetic variants should result in impairment or alteration of the gene expression or function. Functional defects should be reproduced in a model cell line or in a relevant animal model. These also serve as criteria for inclusion into IUIS IEI classification.

Several software to predict whether a given variant is benign/damaging or tolerant/intolerant are available, but this just serves as a predictor [8–11]. Interpretation of genetic data can also be facilitated by using a software such as human gene connectome [12]. This also allows us to have some guidance for subsequent experiments.

It is also important to be able to detect somatic mutations. Immune defects caused by somatic mutation are not, by its definition, “inborn errors of immunity,” but mimic IEI, thus are called as phenocopy of PID. Examples include Ras-associated autoimmune lymphoproliferative disease (ALPS)-like disorder (RALD) and a type of ALPS with somatic FAS mutation. Detection and quantification of the cells with a mutation can also be done by NGS with large reads or with amplicon sequencing/droplet PCR technology once the mutation was detected.

Recent advancements in gene-editing technology, single-cell analysis, iPS cell technology, and model cell line/animal systems enabled us to carry out functional validations relatively easily. With multiparameter flow cytometry or mass cytometry, abundant surface/intracellular information can be obtained with a small number of cells. ChIP-Seq can now be substituted by CUT&RUN or by CUT&TAG that requires far less cells compared to ChIP-Seq. These available and being developed technologies will expedite the discovery of more pathogenic variants in IEI, given the fact that around 2000 genes are implicated in involvement in immune function.

### Molecular pathogenesis of IEI

Hermann J. Muller, a novel laureate in 1946, coined the terms amorph, hypomorph, hypermorph, antimorph, and neomorph to describe the behavior of gene mutation. Another gene behavior is isomorph where the effect of gene mutation is negligible. In general, loss-of-function (LOF) includes antimorph and hypomorph, and gain-of-function (GOF) includes hypermorph, antimorph, and neomorph. Dominant negative gene mutation is categorized into the concept of antimorph, and haploinsufficiency is in hypomorph. There are a few reported cases caused by neomorph, in which mutation of a gene leads to a novel function that non-mutated gene product did not possess [13]. We recently proposed a new concept: heteromeric interference, where a gene product interferes with the function of a partner (often heterodimer forming) molecule. This type of mutation can be identified in a gene variant of a molecule that functions as heterodimer/multimer or forms a complex.

“Muller categorization” is useful when understanding the phenotype and developing therapeutic measures; however, things are often more complicated especially in case one protein functions by forming homodimer, heterodimer, and multimer or by associating with other molecules. It may well be that each missense mutation has different (magnitude and type of) functional consequences and that the mutation could possess more than one function (i.e., negative dominance and neomorph).

In the next two sections, examples of selected IEI that exhibit clinical manifestation mimicking autoimmunity or rheumatic diseases are shown in hope that the molecular basis of the disorders would help in better understanding the adult rheumatic/autoimmune disorders.

### Diseases of immune dysregulation

Studies of monogenic defects in T cell apoptosis, T cell tolerance, or Tregs allowed us to understand self-tolerance and autoimmunity. All the diseases show pathological self-reacting T cell responses, but each category of the defects presents distinctive manifestations and shows distinct clinical phenotype.

Autoimmune regulator (AIRE) is a transcription factor regulating tissue-specific autoantigen presentation in thymic epithelial cells. Mutation in the AIRE gene leads to tissue-specific autoantigens and clinically to protean manifestations of autoimmune disease. The patients typically show type I autoimmune polyglandular syndrome (APS) [14].

FOXP3 is a critical regulator of regulatory T cell (Treg) development and function. Mutation in the FOXP3 gene results in immune dysregulation, polyendocrinopathy, enteropathy, and X-linked (IPEX) syndrome. IPEX patients develop enteropathy, type 1 diabetes, and atopy-like skin disease from an early age and frequently show cytopenia [15]. Other IEI with decreased or dysfunctional Treg show phenotype of IPEX, and these include molecular defects in CD25 (IL-2Ralpha) and CD122 (IL-2Rbeta). All together, they are called tregopathy [5].

CTLA4 is expressed in Treg and is a surface protein that competes with CD28 and inhibits T cell activation. The expression is regulated by FOXP3. CTLA4 haploinsufficiency, a relatively common immune disorder, is characterized by autoimmunity, lymphoproliferation in the lung, and immunodeficiency caused by ultimately exhausted immune system [16, 17].

Autoimmune lymphoproliferative syndrome (ALPS) is a disorder where immune cells do not undergo apoptosis and proliferation because of a defect in a molecule in the FAS/FASL signal pathway. The patients show hepatosplenomegaly, lympho-adenopathy, and autoimmune cytopenia [18].

### Autoinflammatory diseases

Autoinflammatory diseases are usually characterized by fever, skin rashes, or inflammation in the joints or serous membranes. The disorders are not caused by autoimmunity and in many cases are not manifested by susceptibility to infection.

The disorders are classified into three groups, type 1 interferonopathy, inflammasomopathy, and non-inflasommopathy [1].

Type 1 interferonopathy is caused by a mutation leading to overproduction of type I interferon, hyperactivation of interferon-stimulated genes (ISGs), or that of interferon responsive genes (IRGs) [18, 19]. This typically is caused by a mutation in a sensor for extrinsic or intrinsic nucleic acid or by that in an enzyme that cleaves endogenous RNA or DNA. An example of this entity is Aicardi-Goutieres syndrome that is characterized by calcification, demyelination, and brain atrophy [20].

Inflammasomopathy is caused by hyperproduction of IL-1 or IL-18 [21]. The inflammasome is a multiprotein complex that activates caspases to process and secrete IL-1beta and IL-18. One example is pyrin inflammasome, and dysregulated pyrin, which is encoded by *MEFV*, leads to familial Mediterranean fever [22]. Inflamamasomopathy is also caused by a mutation in other inflammasome components or by that in molecules involved in the regulation of inflammasome activation.

The third group of autoinflammatory diseases is mainly caused by the accumulation of signal intermediates that reside downstream of TNF or IL-1 [23]. For instance, a mutation in *TNFAIP3* (A20) that deubiquitinate IκB kinase beta leads to hyperactivation of the NFκB pathway [24], and the patients with *TNFAIP3* mutation exhibit various symptoms resembling Behcet diseases, SLE, or others.

Autoinflammatory diseases and diseases in immune dysregulation are different in that the former is caused by defects in innate immunity and does not show infection episode, while the latter is based on defects in T cell immunity and could present susceptibility to infection. It is of note that these can be found in adult patients with diseases such as Still disease, SLE, Behcet disease, and inflammatory bowel disease. It is thus important to recall and think of IEI when encountering with non-typical rheumatic diseases or autoimmune diseases in childhood as well as in adulthood.

### Our approach towards elucidation of pathogenesis of IEI

Our team has identified causative genes for PID and has worked on the elucidation of the molecular pathogenesis of the disorder and on the development of new therapeutics. Heterologous IKZF mutation in B cell deficiency and autoimmunity [25], heterologous TNFAIP3 mutation in autoinflammation and immunodeficiency [26], heterologous IKBKB variant in common variable immunodeficiency [27], and homozygous TNFSF13 (APRIL) deficiency in plasmacyte defect [28] were reported in the past 5 years.

We are in the process of studying the in-depth pathogenesis of reported and unreported immunodeficiency. We are particularly interested in a disorder caused by a mutation in the IKZF family protein. The IKZF family protein consisted of IKZF1 (IKAROS), IKZF2 (HELIOS), IKZF3 (AIOLOS), IKZF4 (EOS), and IKZF5 (PEGUSUS) [29, 30]. The family protein is essential in the development and differentiation of cells in hematopoietic origin, and the defect in one of the molecules is associated with immunodeficiency (i.e., germline mutation of IKZF1 in B cell defect, combined immunodeficiency, and immunodeficiency with hematological manifestations) [25, 31–33] or with lymphoid malignancy (somatic mutation in IKZF1, IKZF2, or IKZF3 found in acute lymphoblastic leukemia, malignant lymphoma, and chronic lymphocytic leukemia) [34–37].

Another current interest includes OAS1 deficiency. Patients with OAS1 deficiency show hypogammaglobulinemia and pulmonary alveolar proteinosis (PAP) from infancy with defects in B cells and monocytes [38]. The disorder has already been reported, and the identified mutations include A76V, C109Y, and L198V. OAS1 encodes 2′,5′-oligoadenylate synthetase that catalyzes ATP to synthesize 2′,5′-oligoadenyltes (2′, 5′-AS). OAS1 is induced and activated via type I interferon pathway and induced by RNA virus infection. 2′, 5′-AS activates RNAseL that then cleaves and degrades viral RNA [39]. It is of note that all the affected children developed symptoms after respiratory infection, and PAP was associated with the defect of mature alveolar macrophages.

Human OAS consisted of OAS1, OAS2, OAS3, and OASL, and functional differences among the family members are yet to be elucidated. Interestingly, in rodents, the Oas1 family consisted of Oas1a-Oas1h and is robustly activated after type I IFN stimulation [40].

Also, of note is the fact that many SNPs with amino acid substitution are observed in the OAS1 gene in the general population, and some of them are associated with functional defect [41].

To better understand the molecular pathogenesis, we have generated and studied knock-out mice, knock-in mice (in collaboration with Dr. Kakuta at Tokyo University), patient-derived iPS cells, gene-edited disease model iPS cells with a known mutation, monocytes differentiated from the iPS cells, model cell line system, recombinant protein, structural biology approach, and biochemical approach. This inevitably required inter-institutional as well as international collaboration. With this approach, we are hoping to soon delineate whether the mutants harbor loss-of-function, gain-of-function, dominant negative, haploinsufficiency, or neomorphic function. Biochemical and biological natures of nucleic acid sensors or nucleic acid-catalyzing enzymes are yet to be clarified. Interchangeablity of OAS family protein, functional difference of OAS1 isotypes, and other functions of OAS1 other than 2′, 5′-AS are also yet to be explored. There remain more questions than answers.

### The road ahead

It would be surprising if we do not find any more IEI genes in the coming years since there exist approximately 2000 immune-related genes and other seemingly not immune-related gene variants that affect the first-line/second-line defense mechanism to pathogens.

In this article, we introduced a concept of genetic heterogeneity/phenotypic homogeneity (many genes, one phenotype). A mutation in one gene, on the other hand, can show a variety of phenotypes (one gene, many phenotypes). This could be caused by a type and a site of mutation leading to different functions (LOF, GOF, HI, or others). It would also be worthwhile noting that a disease-causing mutation in sibling cases shows different phenotypes. In our ICOS-deficient patients, a sister shows rheumatoid arthritis, psoriasis-like skin region, susceptibility to viruses and bacteria, and hypoimmunoglobulinemia that requires immunoglobulin supplementation [42]. Her brother, on the other hand, does not show susceptibility to infection or autoimmune manifestation in more than 15 years of follow-up period. His IgG is above 700mg/dL with elevated IgM. These cases indicate a presence of modifier genes or an epigenetic factor involved in disease progression. Digenic and trigenic disorders would be identified in the field of IEI. These may serve as a model of multigenic adult immune disorders. Somatic mutation or somatic mosaicism also could exhibit phenotype of IEI requiring quantitative sequencing on different cell types.

IEI is the simplest immune disorder caused by a mutation in a single gene and yet exhibits a wide range of clinical manifestations in a particular disorder.

A more detailed analysis of IEI would certainly lead to a better understanding of more complexed adult immune disorders.

## Conclusions

IEI is a monogenic disorder that displays a variety of symptoms. It is noteworthy that the findings obtained from the research in this field have provided clues to identify molecular pathogenesis of more common disorders. IEI is not rare in a sense that monogenic not rare variants could explain disease susceptibility of common diseases. We are sure to witness results of advances in this field and to add more disease-causing genes in the coming years. Novel technologies will expedite better understanding of the pathophysiology.

## Data Availability

Not applicable

## Abbreviation

AIRE: Autoimmune regulator
ALPS: Autoimmune lymphoproliferative disease
APS: Autoimmune polyglandular syndrome
CVID: Common variable immunodeficiency
EV: Epidermodysplasia verruciformis
HSE: Herpes simplex encephalitis
IEI: Inborn errors of immunity
IPEX: Immune dysregulation, polyendocrinopathy, enteropathy, X-linked
IRGs: Interferon-regulated genes
ISGs: Interferon-stimulated genes
IUIS: International Union of Immunological Societies
PAP: Pulmonary alveolar proteinosis
PID: Primary immunodeficiency
MSMD: Mendelian susceptibility to mycobacterial disease
NGS: Next-generation sequencing
SCID: Severe combined immunodeficiency
